# Effect of home-based interventions on virologic outcomes in adults receiving antiretroviral therapy in Africa: a meta-analysis

**DOI:** 10.1186/1471-2458-14-239

**Published:** 2014-03-07

**Authors:** Nathaniel Chishinga, Peter Godfrey-Faussett, Katherine Fielding, Helen Ayles

**Affiliations:** 1 Zambia AIDS-related TB Project, School of Medicine, P.O Box 50697, Ridgeway campus, Lusaka, Zambia; 2 Department of Clinical Research, London School of Hygiene and Tropical Medicine, Keppel Street, London WC1E 7HT, UK; 3 Department of Infectious Disease Epidemiology, London School of Hygiene and Tropical Medicine, Keppel Street, London WC1E 7HT, UK

**Keywords:** HIV, Virologic outcomes, Home-based interventions, Antiretroviral therapy, Meta-analysis, Sub-Saharan Africa

## Abstract

**Background:**

The success of adherence to combination antiretroviral therapy (ART) in sub-Saharan Africa is hampered by factors that are unique to this setting. Home based interventions have been identified as possible strategies for decentralizing ART care and improving access and adherence to ART. There is need for evidence at individual- or community-level of the benefits of home-based interventions in improving HIV suppression in African patients receiving ART.

**Methods:**

We conducted a systematic review and meta-analysis of the literature to assess the effect of home-based interventions on virologic outcomes in adults receiving ART in Africa.

**Results:**

A total of 260 publications were identified by the search strategy, 249 were excluded on initial screening and 11 on full review, leaving 5 publications for analysis. The overall OR of virologic suppression at 12 months after starting ART of home-based interventions to standard of care was 1.13 (95% CI: 0.51–2.52).

**Conclusions:**

There was insufficient data to know whether there is a difference in HIV suppression at 12 months in the home-based arm compared with the standard of care arm in adults receiving ART in Africa. Given the few trials conducted from Africa, there is need for further research that measures the effects of home-based models on HIV suppression in African populations.

## Background

Successful HIV treatment programmes depend largely on adherence of patients to combination antiretroviral treatment (ART). Several studies have demonstrated that adherence to ART is an important predictor of viral suppression [1-4], antiretroviral resistance [1,5,6], progression to AIDS [1], and death [7,8]. However, there are still challenges of improving adherence to ART in Africa [9]. Home based interventions have been identified as possible strategies of decentralizing ART care and promoting task shifting to improve access and adherence to ART. In this meta-analysis we searched for randomised trials from Africa that used home-based strategies to improve virologic outcomes in patients receiving ART to provide evidence on the feasibility of using this intervention on African populations. The reason for choosing studies from Africa is that unlike developed countries, the success of adherence to ART in sub-Saharan Africa is hampered by factors that are unique to this setting. The magnitude of HIV-related complications is becoming too great for existing clinic infrastructures in Africa. Most public health facilities in sub-Saharan Africa rely on self-presentation of patients to the ART clinic for group counselling on ART adherence and collection of monthly stocks of antiretroviral drugs (ARVs). This strategy risks failing because amidst the rapid scale-up of ART there are severe shortages of suitably skilled health professionals in public health facilities [10].

Existing systematic reviews and meta-analyses on home-based interventions [11] and directly observed therapy for ART [12,13] have few studies from Africa. There are significant new data from trials in Africa on which to update these reviews. We perform a meta-analysis on the effect of home-based interventions on virologic outcomes in adults receiving ART in Africa.

## Methods

### Eligibility criteria

Articles were restricted to English language and included in this meta-analysis if they:

1. were randomised controlled trials (RCT) or cluster randomised trials (CRT) of patients receiving ART, because these are considered the gold standard for assessing the effects of an intervention;

2. included virologic outcomes, and

3. reported on home-based interventions in Africa that used either family or lay and/or professional people to provide all forms of treatment, care or support in the HIV-infected person’s home, as compared with hospital or health facility-based care; even though (i) patients still had to visit the clinic from time to time and (ii) the clinic remained responsible for the prescription of anti-retroviral regimen and changing the therapy.

### Search methods for identification of studies

The following data sources were searched in May 2010, September 2011 and the search repeated in December 2012 for updates: PubMed/Medline from 2001 to 2012 and EMBASE from 2001 to 2012. Additional studies not indexed in PubMed/Medline and EMBASE were searched from abstracts presented at the Conference on Retroviruses and opportunistic Infections (CROI) from 2001 to 2012 and the International AIDS Society/International AIDS (IAS/AIDS) conferences from 2000 to 2012. Also, cross references were searched from the published systematic reviews and meta-analyses discussed above.

The syntax and search terms were adapted from the “Cochrane Highly Sensitive Search Strategy for identifying randomised trials in PubMed/Medline: sensitivity-maximizing version (2008 revision)” filter [14]. The search terms for detecting randomised studies in EMBASE were adapted from Wong and colleagues’ “Best specificity terms” [15] (Table 1).

**Table 1:** Database search terms

**Database**	**Terms**
PubMed/Medline	1. Randomized controlled trial.pt.
	2. Controlled clinical trial.pt.
	3. Randomized.ab.
	4. Placebo.ab.
	5. Drugtherapy.fs.
	6. Randomly.ab.
	7. Trial.ab.
	8. Groups.ab.
	9. 1 or 2 or 3 or 4 or 5 or 6 or 7 or 8
	10. Exp animals/not humans.sh.
	11. 9 not 10
	12. (cluster$ adj6 randomi$).mp.
	13. ((communit$ adj6 intervention$) or (communit$ adj6 randomi$)).mp.
	14. group$ randomi$.mp.
	15. cluster-randomi$.mp.
	16. 12 or 13 or 14 or 15
	17. 11 or 16
	18. ((direct$ adj6 observ$) or (treat$ adj6 partner$) or (treat$ adj6 support$) or (patient$ adj6 support$) or (patient$ adj6 select$) or (peer adj6 health$) or (peer$ adj6 deliver$)).mp.
	19. (Home based care or (facilit$ adj6 base$)).mp.
	20. 18 or 19
	21. Exp antiretroviral therapy, highly active/
	22. Hiv treatment.mp.
	23. Hiv care.mp.
	24. 21 or 22 or 23
	25. ((viral load) or (HIV viral load) or (virologic$ adj6 fail$) or (virologic$ adj6 outcome$) or (treat$ adj6 fail$) or (treat$ adj6 success$)).mp.
	26. 24 or 25
	27. 20 and 26
	28. 17 and 27
	29. Exp africa/
	30. 28 and 29
EMBASE	1. Double-blind:.mp. or placebo.tw. or blind:.tw.
	2. (Cluster: adj6 randomi:).mp.
	3. ((communit: adj6 intervention:) or (communit: adj6 randomi:)).mp.
	4. Group: randomi:.mp.
	5. Cluster-randomi:.mp.
	6. 2 or 3 or 4 or 5
	7. 1 or 6
	8. ((direct: adj6 observ:) or (treat: adj6 partner:) or (treat: adj6 support:) or (patient: adj6 support:) or (patient: adj6 select:) or (peer adj6 health:) or (peer: adj6 deliver:)).mp.
	9. (Home based care or (facility: adj6 base:)).mp.
	10. 8 or 9
	11. Exp antiretroviral therapy, highly active/
	12. Hiv treatment.mp.
	13. Hiv care.mp.
	14. 11 or 12 or 13
	15. ((viral load) or (HIV viral load) or (virologic: adj6 fail:) or (virologic: adj6 outcome:) or (treat: adj6 fail:) or (treat: adj6 success:)).mp.
	16. 14 or 15
	17. 10 and 16
	18. 7 and 17
	19. exp africa/
	20. 18 and 19

### Data collection and analysis

#### Selection of studies

Search results were merged using EndNote X3 (Thomson Reuters, TX, USA) reference manager, and duplicate records removed. The titles and abstracts of the articles were then examined and reports that were not randomised studies and those that were not relevant were removed. Full-texts that were potentially relevant were then examined for compliance with eligibility criteria. Studies that did not meet the inclusion criteria were excluded; the remaining studies were included and data collection done. Information obtained for each study included:

1. Study design (RCT or CRT)

2. The setting (primary or tertiary level of care) and type of intervention (including frequency and duration of the intervention) vs. the standard care at the clinic.

3. The sample size of each trial

4. Characteristics of the trial participants (including mean age distribution, gender and marital status)

5. Virologic outcomes.

### Data extraction and management

The data was extracted following the search terms. The outcome of interest for this meta-analysis was HIV suppression (dichotomous outcome) in adult Africans at 12 months after starting ART. Summary data was collected for each intervention group in a study and entered in STATA version 11 (STATA corp., Texas, USA).

### Assessment of risk of bias in included studies

The risk of bias (methodological quality) of the extracted studies was assessed using the Cochrane Collaboration’s ‘Risk of bias’ tool [14]. This checks whether the trials reported on sequence generation, allocation of concealment, blinding, incomplete outcome data of patients lost to follow-up (i.e., missing at follow-up as equal to virologic failure) and other biases. The following were considered as other biases: (i) recruitment bias; (ii) baseline imbalances. In addition, the following were assessed in CRTs (iii) loss of clusters; and (iv) incorrect analysis; and comparability with individually randomised trials [14]. In CRTs, individual patient assignment of the intervention was assumed to be by the allocation of the intervention to the cluster in which the individuals resided. A ‘Risk of bias’ graph was then produced using RevMan version 5.0 (Copenhagen: The Nordic Cochrane Centre, the Cochrane Collaboration, 2008).

### Measures of treatment effect and unit of analysis

The measure of effect is the odds ratio (OR) of HIV suppression in the home-based to the health facility-based group. If the data in the articles was measured using rate ratios, the rate ratio was used to estimate the OR [16]. In order to combine the effects of RCT and CRT, the OR of HIV suppression and the 95% confidence interval (CI) were used to calculate the log odds ratio (log_e_ OR) and its standard error (SE (log_e_OR)). Because the unit of allocation in CRTs is a cluster, the *cluster-adjusted risk ratio* and *rate ratios* from each CRT were used as estimates of *cluster-adjusted OR* and the log_e_ OR and the SE (log_e_ OR).

### Assessment of heterogeneity

The between-study heterogeneity variance of the log_e_ ORs, tau-squared (I^2^), was used to measure heterogeneity between trials. An I^2^ ≥ 30% and χ^2^ p-value < 0.10 were used test for evidence of heterogeneity [14]. Also, a visual check of confidence intervals of individual studies (depicted by horizontal lines on the forest plot) was used; if the confidence intervals poorly overlapped, this would indicate presence of heterogeneity.

### Assessment of reporting biases

A ‘funnel plot’ of standard error vs. effect size estimate of the intervention on virologic suppression was created to visually assess for asymmetry as an indication of publication bias.

### Data synthesis

The log_e_ OR and SE (log_e_ OR) for the RCTs and CRTs were combined using the inverse-variance method for meta-analysis in **metan** (STATA corp. version 11, Texas, USA) to produce a forest plot. An OR > 1 indicated HIV suppression favouring the home-based group. All P-values were two sided; at 5% significance level (except in the testing for heterogeneity where a χ^2^ p-value of 10% was used as discussed above).

### Sensitivity analyses

As *a priori*, a sensitivity analysis was going to be performed only when there was evidence of heterogeneity between studies. The sensitivity analysis would be performed by including and excluding studies based on indicators in the Cochrane Collaboration’s ‘Risk of bias’ tool listed above of reporting (i) sequence generation, (ii) allocation of concealment, (iii) blinding, (iv) incomplete outcome data of patients lost to follow-up (i.e., missing at follow-up as equal to virologic failure) and (v) other biases.

## Results

### Description of studies

**Figure 1:**
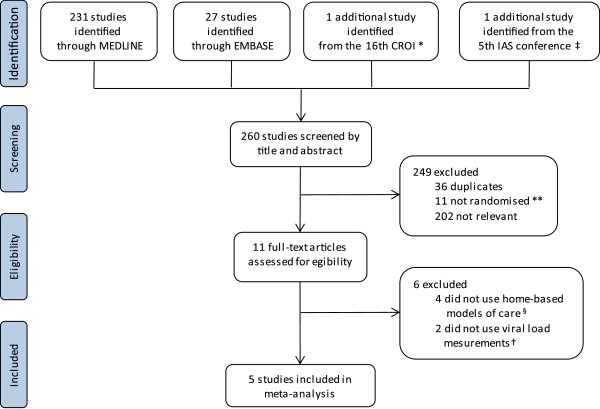
**Flow of articles selected for meta-analysis; adapted from the PRISMA statement.** CROI, Conference on Retroviruses and Opportunistic Infections. IAS, International AIDS Society/International AIDS Conference on HIV Pathogenesis, Treatment and Prevention. *Matovu, 2011 [17]. **Konate, [18]; Gusdal, [19]; Igumbor, [20]; Nglazi, [21]; Kipp, [22]; Wouters, [23]; Wouters, [24]; Shacham, [25]; Idoko, [26]; Nachega, [27]; Weidle, [28]. ‡ Naidoo, [29]. § Lester, [30]; Sarna, [31]; Fairall, [32]; Naidoo, [29]. † Stubbs, [33]; Pearson, [34].

There were 260 articles identified after using the two search methods described above. These 260 articles included the trials from Africa identified in the earlier meta-analyses stated above. Out of these 260 articles, 249 were excluded after screening by title and abstract. The remaining 11 articles were assessed for eligibility by reading through the full-text; 5 articles were found to meet the inclusion criteria for this meta-analysis (Figure 1).

### Characteristics of included studies

Five randomised trials were included; three were RCTs [17,35,36] and two CRTs [37,38] (Table 2). One study was conducted in a Prevention of Mother to Child Transmission of HIV (PMTCT) programme with women only who were attending ante-natal clinic while the rest of the studies included men and women. All five trials reported on the effect of a home-based intervention on virologic outcomes in adults at 12 months (48 weeks) of starting ART. The duration of the studies ranged from 12 months to 36 months. The number of participants analysed in each study ranged from 85 to 1 212. The combined sample size of patients analysed was 2 688; 1 614 patients were in the intervention (home-based) arm and 1 074 patients in the control (standard- , facility-, clinic-based) arm.

**Table 2:** Characteristics of included studies

**Study**	**Design**	**Study duration**	**Setting**	**Inclusion and exclusion criteria**	**Age**	**Gender**	**Sample size analysed**	**Intervention**	**Interval to viral load measure**	**HIV viral load outcomes**
Jaffar et al. [37]	Cluster randomised trial: **Equivalence trial**	36 months	Home and Outpatient clinics in Jinja district, Uganda	**Included:** 1. Patients >18 years old; 2. Starting ART for the first time. **Excluded:** Patients living 100 km away from the ART clinic, where the provision of the home-based intervention was not possible.	Mean age in intervention arm 37 years (range 32–44); Mean age in control arm 38 years (range 33–44)	73% Female (625/859) in intervention arm; 68% Female (406/594) in control arm	**n = 1212:** 729 (22 clusters) in intervention arm; 483 (22 clusters) in control arm	**Intervention arm:** monthly visits to a patient’s home by field staff to deliver ARVs and monitor signs and symptoms of drug toxicity or disease progression, and provide adherence support. **Control arm:** Standard care provided at the clinic.	Viral load measured at 6, **12**, 18, 24,30 and 36 months	**Time to RNA viral load >500 copies/ml.** “Home-based HIV care was as effective as was facility-based care.”% *of persons with undetectable viral load at 12 months in intervention arm = (729–117)/729 = 84.0%;% of persons with undetectable viral load at 12 months in control arm: (483–80)/483 = 83.4%.* Rate ratio 1 · 04 (95% CI: 0 · 78 – 1 · 40; equivalence shown). **Odds Ratio = 1.04 (95% CI: 0.78 – 1.40)**^**§**^
Nachega et al. [35]	Randomised controlled trial: **Superiority trial**	24 months	Home and Public clinic in Cape Town, South Africa	**Included:** 1. Male or non-pregnant female ≥18 years old; 2. HIV infection documented by two serologic tests; 3. Eligible to start ART (CD4 ≤ 200 cells/μL or WHO Clinical Stage IV disease); 4. Living in the study site catchment area at a stable address; 5. Willing to disclose HIV status to a treatment supporter; 6. Signed informed consent. **Excluded:** 1. Patients with prior ART use; 2. Life expectancy <6 months; 3. Karnofsky Performance Score <60; 4. Serious liver disease; or 5. History of single dose nevirapine for prevention of mother to child transmission of HIV infection.	Mean age in intervention arm 35.7 years (SD 9.7); Mean age in control arm 36.7 years (SD 9.2)	57.7% female in intervention arm; 57.7% female in control arm	**n = 272:** 136 in intervention arm; 136 in control arm	**Intervention arm:** In addition to standard care at the clinic, trained treatment supporters provided ART adherence support, observed at least one medication dose daily and documented it on a study adherence chart. **Control arm:** Standard care provided at the clinic.	Viral load measured at **12** and 24 months	**RNA viral load <400 copies/ml**. “DOT-ART showed no effect on virologic outcomes”.% *of persons with undetectable viral load at 12 months in intervention arm = 99/(99 + 37) = 72.8%;% of persons with undetectable viral load at 12 months in control arm = 93/(93 + 43) = 68.4%.***Odds Ratio of HIV suppression at 12 months =** (99/37)/ (93/43) = **1.24 (95% CI: 0.73 – 2.09)**
Chang et al. [38]	Cluster randomised trial: **Superiority trial**	48 months	Home and Public clinics in rural Rakai District, South West Uganda	**Included:** All adult patients who were either already on ART at the start of the trial or were started on ART at any time during the trial.	Mean age in intervention arm 35.5 years (range 15–76); Mean age in control arm 34.0 years (range 17–70)	65.8% female in intervention arm; 67.5% female in control arm	**n = 620:** 456 (10 clusters) in intervention arm; 164 (5 clusters) in control arm	**Intervention arm:** Standard care of ARV delivery at the clinic plus biweekly home-based review by a peer health worker who checked for symptoms of treatment failure; patient self-report of adherence; pill count and provision of counselling and education in ART adherence and general HIV/AIDS-related issues. **Control arm:** Standard ART care provided at the clinic.	Viral load measured at 6, **12**, 18, 24,30, 36, 42, 48 months	**RNA viral load >400 copies/ml.** At 12 months (48 weeks), no significant differences were found between the intervention and control arm.% *of persons with undetectable viral load at 12 months in intervention arm = (456–42)/456 = 90.8%;% of persons with undetectable viral load at 12 months in control arm = (164–18)/164 = 89.0%*. Risk Ratio 0.83 (95% CI: 0.47 – 1.48). **Odds Ratio of HIV suppression at 12 months =** 1/0.83 = **1.20 (95% CI: 0.68 – 2.13)**
Taiwo et al. [36]	Randomised controlled trial: **Superiority trial**	12 months	Home and Tertiary Hospital HIV clinic in Jos, Nigeria	**Included:** 1. HIV-1-infected; 2. Treatment-naïve; 3. Age >15 years; 4. Eligible for ART (clinical diagnosis of AIDS, CD4 count <350 cells/μL with HIV-related symptoms or CD4 count <200 cells/μL regardless of symptoms); 5.Willingness and ability to select a treatment partner. **Excluded:** Patients with severe illness.	Mean age 34.2 years (SD 8.9)	66.1% female in intervention arm; 63.3% in control arm	**n = 499:** 248 in the intervention arm; 251 in the control arm	**Intervention arm:** In addition to standard care at the clinic, a treatment partner residing in same house or close proximity observed the ingestion of ARVs at least once daily. The treatment partner reported adverse effects and reminded participants of drug pick-up at the hospital. **Control arm:** Standard care provided at the Hospital HIV clinic.	Viral load measured at 6 and **12** months	**RNA viral load <400 copies/ml.** At 12 months (48 weeks), no significant differences were found in HIV suppression between the intervention and control arm. % *of persons with undetectable viral load at 12 months in intervention arm = 162/248 = 65.3%;% of persons with undetectable viral load at 12 months in control arm = 149/251 = 59.4%.***Odds Ratio = 1.28 (95% CI: 0.89 – 1.84)**
Matovu et al. [17]	Randomised controlled trial: **Non-inferiority trial**	12 months	Home and PMTCT follow-up clinic, Mulago National Referral Hospital in Kampala, Uganda	**Included:** 1. Female; 2. Age ≥18 years; 3. Provision of written informed consent; 4. ≥36 weeks of gestation; 5. Eligible for ART (WHO Clinical Stage II/IV or CD4 counts <200 cells/μL); 6. Demonstrated compliance with ART screening visits; 7. Residence in a stable home within 15 km of Mulago Hospital; 8. Willing and able to come to the clinic regularly without transport reimbursement; 9. Willing to be home visited.	Mean age in intervention arm 27.8 years (SD 4.9); Mean age in control arm 27.0 years (SD 5.4)	All Females	**n = 85:** 45 in the intervention arm; 40 in the control arm	**Intervention arm:** Involved peer counsellors and home visiting, combined with nurses providing care at routine visits, and longer intervals between scheduled visits. **Control arm:** Standard care provided at the Hospital promoted adherence through routine counselling at each scheduled visit, care provided by a medical officer at each visit, and shorter intervals between visits.	Viral load measured at 6 and **12** months	**RNA viral load <400 copies/ml.**% *of persons with undetectable viral load at 12 months was similar in the intervention (= 88%) and control (= 91%) arm.***Odds Ratio of HIV suppression at 12 months =** (0.88/ (1–0.88)) / (0.91/ (1–0.91)) = **0.73 (95% CI: 0.18 – 2.96)**

^§^Jaffar et al. [37]: The rate ratio obtained from this trial was based on time to virologic failure measured over a period of 36 months in contrast to the other studies included in this search that used risk ratios or odds ratios (OR) at 12 months. This rate ratio was reciprocated so as to change it from time to failure to time to HIV suppression. The rate ratio was reciprocated again so that the ratio is of home-based to facility-base care. Finally this home-based to facility-based care rate ratio was used to approximate the OR of HIV suppression, which resulted in the same estimate OR of 1.04 (95% CI: 0.78 – 1.40).

### Risk of bias in included studies

The methodological qualities of the included trials were assessed using the ‘Risk of bias’ tool (Table 3). One study reported on blinding of study personnel, though this was only restricted to the study pharmacist. It is not clear in the four other studies whether laboratory personnel measuring the viral load outcomes were blinded. Three studies excluded missing data from the analysis of virologic outcomes. Two studies were not free of other problems that could put them at risk of bias (trial terminated early and contamination).

**Table 3:** Risk of bias in included studies

**Study**	**Adequate sequence generated?**	**Allocation concealment?**	**Blinding of participants and personnel?**	**Blinding of viral load assessment?**	**Incomplete outcome data addressed? (Missing at follow up = treatment failure)**	**Free of other bias?**
Jaffar et al. [37]	Yes.	Yes.	No.	Yes.	No.	Yes.
	Cards were sealed in advance and labelled with stratum numbers and placed into a box	Sealed cards were drawn from a concealed box.	The study was cluster randomized trial; researchers and participants knew which clusters were receiving the interventions.	Probably done, because Blood taken for viral load testing was for research purposes; the testing was done in batches rather than in real-time.	“Those who withdrew or were lost to follow-up before 12 months were excluded from the primary endpoint analysis…”	The two groups were well balanced according to baseline characteristics apart from CD4-cell count, which was lower in the home-based than in the facility-based group [39]. There were no losses of clusters. The analysis adjusted for the effect clustering
Nachega et al. [35]	Yes.	Yes.	No.	Unclear.	Yes.	No.
	Probably done, because there was sequential allocation concealment	“[T]reatment assignments were placed in opaque envelopes, which were sequentially opened by the study coordinator at enrolment.”	The study was an open-label, randomized controlled trial; both the researchers and the participants knew which intervention was being administered.	Measurement of viral loads performed was every 6 months as part of routine monitoring	Missing viral load values were considered detectable.	Trial terminated early for futility by an independent Data and Safety Monitoring Board
Chang et al. [38]	Yes.	Yes.	No.	Unclear.	No.	No.
	An allocation sequence was generated	Random allocation was by drawing of lots.	The study was cluster randomized trial; researchers and participants knew which clusters were receiving the interventions.	"Viral loads … were performed every 24 weeks on all patients as part of routine patient monitoring procedures."	Those who died or were lost to follow-up were excluded from the analysis of shorter-term virologic outcomes	Contamination in the control arm was reported in subsequent evaluation study [40].
Taiwo et al. [36]	Yes.	Yes.	Yes.	No.	Yes.	Yes.
	“Using a computer-generated allocation sequence, randomization was performed …”	Probably done, because there was computer-generated allocation sequence.	“The study pharmacist, who was blinded to treatment arm, provided one-on-one reinforcement of the education provided by the adherence counsellor plus information specific to each participant’s regimen.”	Probably not done because patients who had detectable viral loads at week 24 underwent intensive adherence retraining with the adherence counsellor.	“[P]articipants who were missing virologic indicators and were reported to have died were counted as failures.”	There were no significant differences between treatment groups at baseline.
Matovu et al. [17]	Yes.	Unclear.	No.	Unclear.	No.	No.
	Probably done, because patients were randomly allocated.	Insufficient information to permit judgement of “Yes” or “No”.	The study was an open randomized non-inferiority intervention trial.	Insufficient information to permit judgement of “Yes” or “No”.	Patients lost to follow-up were excluded from the analysis.	Baseline viral load was adjusted for. This means there were significant differences in viral loads between intervention groups at baseline.

**Figure 2:**
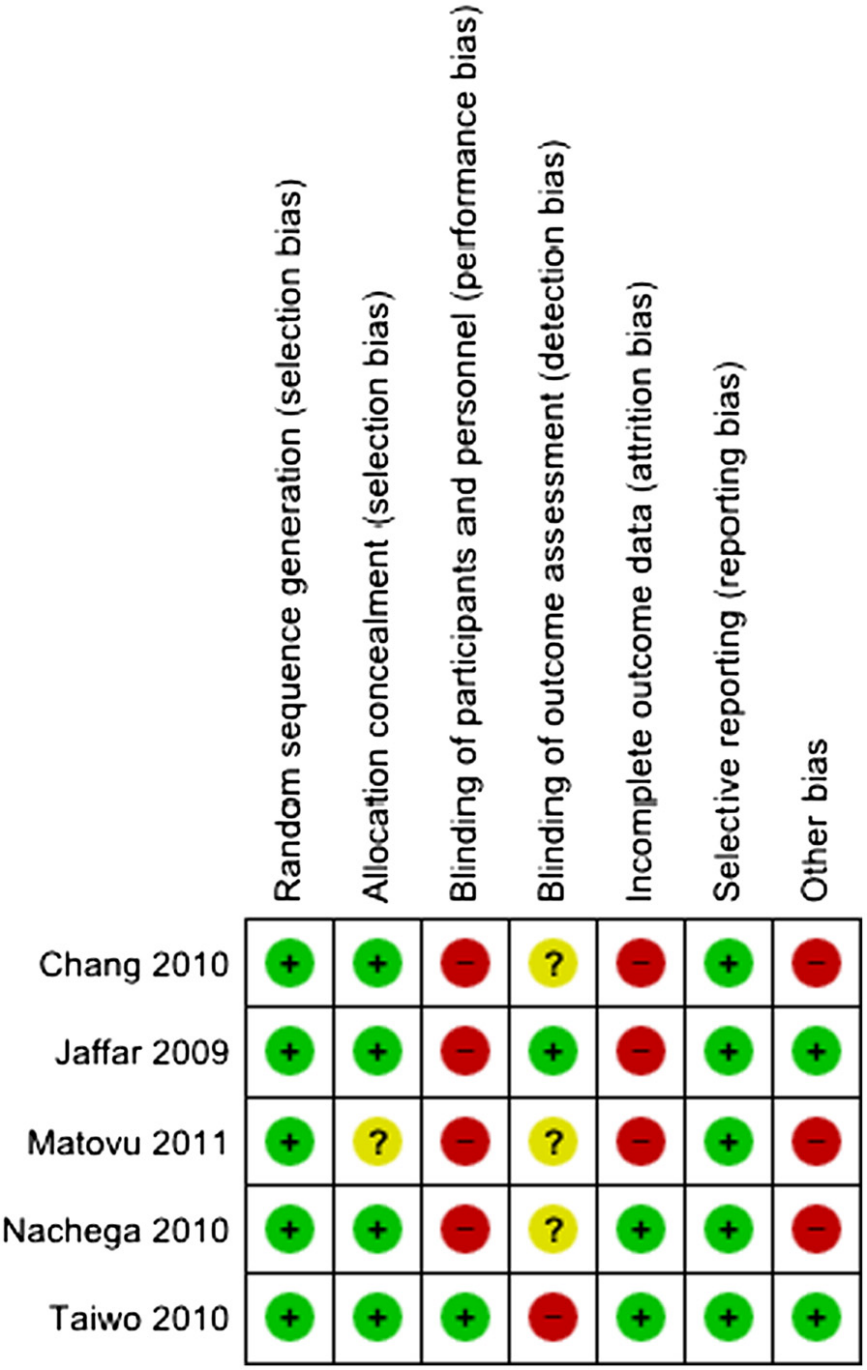
Summary of risk of bias: judgement on each included study.

The study by Matovu and colleagues showed the highest risk of bias because it failed to meet most of the criteria, including lack of information on adequate allocation of concealment and blinding of outcome assessment. It also showed high risk of bias on blinding of participants and personnel; incomplete outcome data of patients lost to follow-up was excluded from the analysis, and ‘other biases’ of baseline imbalance in viral loads between the intervention groups (Figure 2).

**Figure 3:**
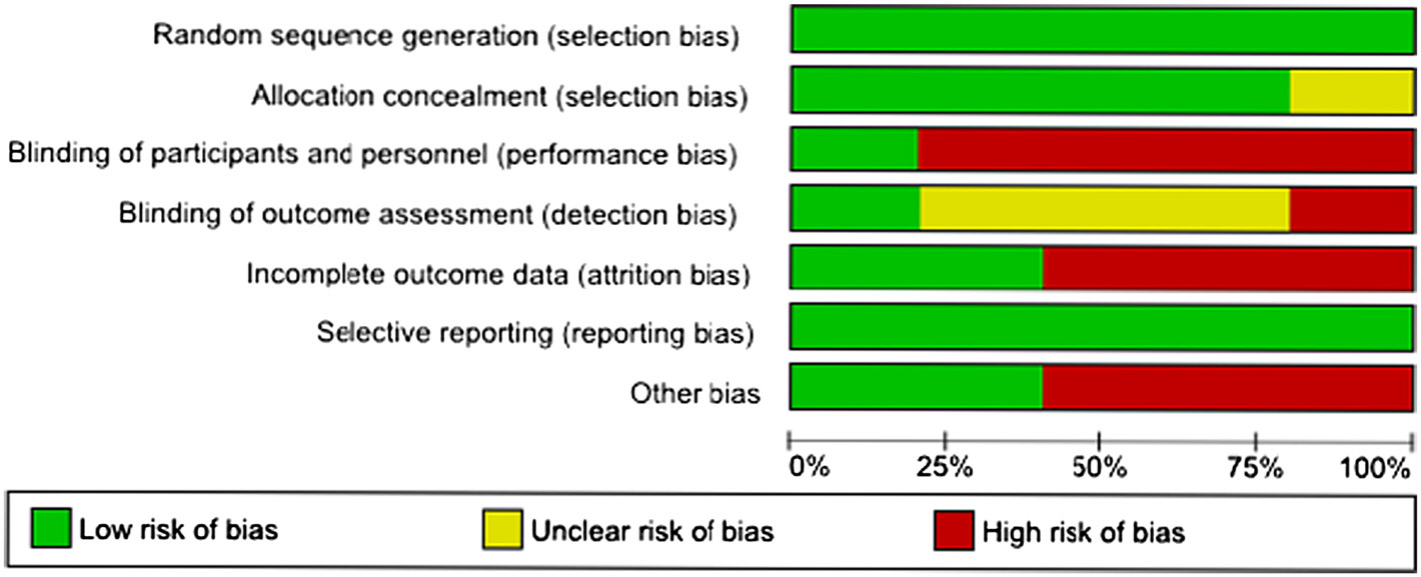
Graph of risk of bias: judgement about each risk of bias item presented as percentages across all included studies.

The proportion of studies with each of the judgements (‘Yes’, ‘No’, ‘Unclear’) for each entry in the ‘Risk of bias’ tool were examined (Figure 3). The risk of bias in the included studies was highest from lack of blinding of participants and study personnel, and lowest from adequate random sequence generation and free of selective reporting.

### Effect of intervention

**Figure 4:**
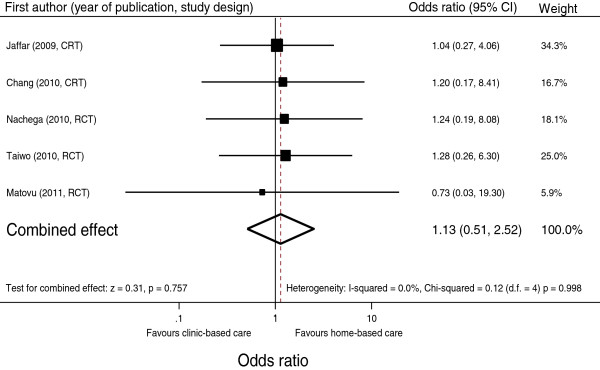
**Odds ratios of home-based to health facility-based HIV care of HIV suppression.** The trials were arranged according to study design; CRT, cluster randomized trial; RCT, Randomized controlled trial.

The overall OR at 12 months of starting ART of home-based to standard-based care was 1.13 (95% CI: 0.51 to 2.52, p = 0.757) (Figure 4).

The I^2^ showed no heterogeneity between trials (I^2^ = 0.0%, p = 0.998). Therefore no sensitivity analyses were performed to assess differences in the studies.

### Detecting publication bias

**Figure 5:**
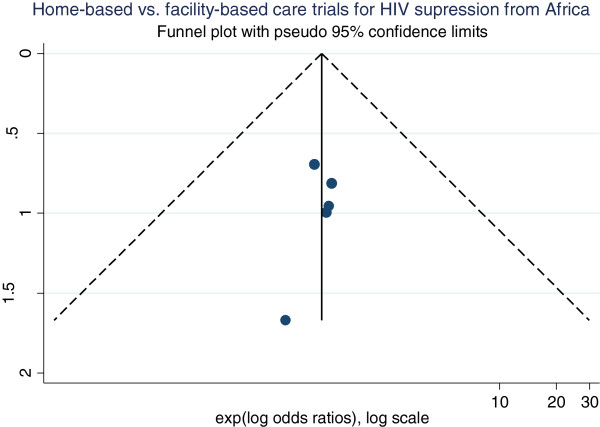
**Funnel plot of five randomised trials comparing home-based interventions with health facility-based care in Africa.** The vertical line in the funnel plot represents the fixed effects summary estimate (using inverse-variance weighting), while the sloping lines represents the expected 95% confidence intervals for a given standard error (assuming no heterogeneity between studies).

A funnel plot was used to ascertain publication bias (Figure 5). The plot was symmetrical indicating the absence of publication bias. The effect of the small study by Matovu and colleagues [17] is at the bottom of the graph showing a wide spread, while the effect of the larger study by Jaffar and colleagues [37] is at the top of the graph showing a narrow spread (increased precision). The effects of all five studies are close to the true intervention odds ratio of 1.13.

No statistical tests for funnel plot asymmetry were performed because at least 10 studies are required to be included in the meta-analysis for these tests to be performed. When there are few studies, the power of the tests is too low to distinguish chance from real asymmetry [14].

## Discussion

### Summary of results

Neither superiority nor non-inferiority of the home-based interventions compared with standard of care has been clearly demonstrated with existing interventions to date. Based on the overall 95% confidence interval in this meta-analysis, it is conceivable that the effect of the home-based care does lead to an almost 2-fold worse outcome of unsuppressed HIV (OR 0.51) or that it is a lot better than the clinic-based care (OR 2.52) - but this meta-analysis is underpowered (by the few studies included) to demonstrate superiority of the home-based over the clinic-based care model. In addition, the home-based model in practice depends on clinic-based activities (including laboratory tests, monitoring of drug toxities and changing of ART regimen). Despite one study (by Jaffar and colleagues) being an equivalence trial, another (by Matovu and colleagues) being a non-inferiority trial and the other three being superiority trials, this did not affect the overall result (i.e. no evidence of heterogeneity).

### Overall completeness and applicability of evidence

The studies included in this meta-analysis were conducted in sub-Saharan countries; where the challenge of shortages of health workers and increased demand for care at the clinic is apparent. Home-based interventions for ART may still have the potential of complementing existing health facility-based care in reducing the burden of HIV care. Home based interventions can potentially be delivered with minimum laboratory tests at the clinic. This is substantiated by a randomised non-inferiority trial conducted in Uganda and Zimbabwe that demonstrated that ART can still be delivered safely without routine laboratory monitoring at the clinic of toxic ART effects [41].

However, for home-based interventions to be successfully rolled out in an African setting, there would still be need for long-term management of HIV patients using culturally acceptable approaches that promote adherence to ART; that can also be easily integrated into public health models of ART care [42]. As demonstrated by Chang and colleagues in 2009 and 2010 [38,43] and recently by Arem and colleagues in 2011 [40], this can be achieved through training of HIV patients (nominated by fellow patients) to provide HIV care in homes. Consequently, this may provide long-term benefits of adherence to ART and retention in care [37].

Jaffar and colleagues in 2009 demonstrated that the costs of health services for ART care is similar in the home-based intervention and the health facility-based group (US$793 in the home-based intervention group and US$838 in the health facility-based group) [37]. However, for the home-based intervention to be sustainable, there is need for ongoing support, training and consistent remuneration for the home-based workers from existing health systems [44].

### Quality of the evidence

Only five trials were found to have met the study inclusion criteria and the combined sample size analysed was 2 688. Each study was assessed for its methodological limitation. The study by Matovu and colleagues [17] showed the highest risk of bias due to failure of this study to meet most of the criteria in the ‘Risk of bias’ assessments.

Two [35,36] of the five studies included used intention-to-treat analyses with losses to follow-up treated as virologic failures. Like most prospective studies, losses-to-follow-up can be a source of selection bias; as it is *not* known what the true outcome of those lost to follow-up is i.e. whether they died as a result failing treatment or as a result of another competing cause of death or were still alive at the end of follow-up but moved out of study site and were thus not available for measuring the outcome at the end of the study. With such losses to follow-up it was hard to ascertain with certainty what the true outcomes were at study completion for the patients in these trials. For example, in the trial by Taiwo and colleagues, 14 out of 248 in the home-based group and 34 out of 251 in the health facility-based group were not found at the end of the study and were treated as failures [36]. These 14 losses in the home-based group and 34 in health facility-based group might have influenced the results differently in the Taiwo and colleagues’ study. Also, after an evaluation of the CRT by Chang and colleagues [38], Arem and colleagues [40] contended that direct and indirect contamination in the health facility-based arm could have reduced the ability of the CRT to detect home-based intervention effects, and may explain why no differences were seen in early virologic outcomes between arms or in cumulative risk of virologic outcomes. Direct contamination occurred when some patients in the health facility-based arm started volunteering to take up tasks that were similar to those of the peer health workers in the home-based intervention arm. Indirect contamination occurred through task shifting which resulted in overall gains in the quality of the ART program during the study period [40].

### Potential biases in the review process

The search for studies was performed in MEDLINE and EMBASE. Additional articles were identified from abstracts presented at the CROI and IAS conferences. It is likely that all relevant articles from Africa were identified and that there are few randomised studies that have been conducted in Africa on the effect of home-based interventions on virologic outcomes in patients receiving ART.

### Agreements and disagreements with other studies or reviews

The meta-analysis by Ford and colleagues [12] also found a lack of effect of the directly observed ART intervention; the risk ratio was 1.04 (95% CI: 0.91 to 1.20). As noted by Hart and colleagues [13] this effect estimate in the study by Ford and colleagues was smaller than their finding of RCTs despite also not being significant; risk ratio 1.18 (95% CI: 0.99 to 1.42). This was also the case in this meta-analysis where we found the overall risk OR to be higher than that of Ford and colleagues’, though with wider 95% confidence intervals; risk OR 1.13 (95% CI: 0.51 to 2.52). The reason for this difference may be that Ford and colleagues included estimates from the post intervention period; during which the efficacy of the intervention may have waned [13], unlike in this meta-analysis where the duration considered for the analysis was shorter .i.e., 12 months from starting ART. If the effects of directly observed ART intervention are not durable .i.e., once people graduate from the directly observed ART intervention there is no benefit on adherence to ART, there may be need for directly observed ART interventions that are repetitive [45,46] or ongoing [47].

## Conclusions

There was insufficient data to know whether there is a difference in HIV suppression at 12 months in the home-based arm compared with the standard care arm in adults receiving ART in Africa. However, given the high shortages of health workers in public health facilities in Africa, home-based interventions for ART, in addition to other approaches that have been proven to be effective such as use of mobile phone short message service [30,48] and use of “buddies” to provide adherence support [49], may complement existing health facility-based care in reducing the burden of care. By task shifting HIV care to peer workers in the community that visit, provide ARVs and witness the dosing of ARVs, adherence can be promoted and this *would not* lead to poorer virologic response to ART in African settings.

Despite a thorough literature search, only five studies were identified to have met the inclusion criteria in this meta-analysis. This shows that there are few randomized trials on home-based interventions on HIV suppression that have been conducted and reported in Africa. There is need for further research in Africa that measures the effects of home-based models on HIV suppression at population level.

## Competing interests

The authors declare that they have no competing interests.

## Authors’ contributions

NC, HA, PGF and KF were involved in the conception of the study. NC did the literature search, reviewed the titles, abstract and full-text, and performed the analysis. NC wrote the first draft of the manuscript and all authors revised it critically for intellectual content. All authors gave final approval of the version to be published.

## Pre-publication history

The pre-publication history for this paper can be accessed here:

http://www.biomedcentral.com/1471-2458/14/239/prepub
